# Popliteal lymph node dissection for metastases of cutaneous malignant melanoma

**DOI:** 10.1186/1477-7819-12-135

**Published:** 2014-05-01

**Authors:** Frederico Teixeira, Vitor Moutinho, Eduardo Akaishi, Gabriella Mendes, Andre Perina, Tiberio Lima, Margareth Lallee, Sergio Couto, Edivaldo Utiyama, Samir Rasslan

**Affiliations:** 1 Department of General Surgery, Surgical Oncology Group, University of Sao Paulo School of Medicine, Sao Paulo, Brazil

**Keywords:** Lymph nodes, Malignant melanoma, Metastases, Operative technique, Popliteal fossa, Surgery

## Abstract

Popliteal lymph node dissection is performed when grossly metastatic nodal disease is encountered in the popliteal fossa or after microscopic metastasis is found in interval sentinel nodes during clinical staging of cutaneous malignant melanoma. Initially, an S-shaped incision is made to gain access to the popliteal fossa. A careful *en bloc* removal of fat tissue and lymph nodes is made to preserve and avoid the injury of peroneal and tibial nerves as well as popliteal vessels, following the previous recommendations. This rare surgical procedure was successfully employed in a patient with cutaneous malignant melanoma and nodal metastases at the popliteal fossa. The technique described by Karakousis was reproduced in a step-by-step fashion to allow anatomical identification of the neurovascular structures and radical resection with no post-operative morbidity and prompt recovery. Popliteal lymph node dissection is a rarely performed operative procedure. Following a lymphoscintigraphic examination of the popliteal nodal station, surgeons can be asked to explore the popliteal fossa. Detailed familiarity of the operative procedure is necessary, however, to avoid complications.

## Background

Metastasis to the lymph nodes represents the most important predictor of disease outcome among patients with malignant melanoma (MM) of the skin [1]. Usually, metastasis from MM in the distal leg and foot primarily affect the lymph nodes in the inguinal nodal basins. In some patients, however, there is direct drainage to popliteal lymph nodes, although this is an infrequent event and rarely reported in the literature [2].

When metastatic disease is identified in the popliteal fossa, surgeons are called on to explore the popliteal nodal station and perform radical lymphadenectomy. This necessitates a profound familiarity of the surgical anatomy and the technique of this uncommon operative procedure.

In our clinic, this procedure was recently performed in a patient with MM of the heel, presented with concomitant popliteal and inguinal lymph node metastases. In this communication, a detailed description of the surgical anatomy is provided and the technique of the popliteal lymph node dissection is described.

## Case presentation

The patient (JRS), a 58-year-old man was subjected to an excisional biopsy of a dark skin lesion in the left foot. On light microscopy, an acral melanoma was diagnosed, with a Breslow thickness of 1.5 mm. Clinical staging was T2 N0 M0, with a normal LDH (355 mg/dL) and normal chest and abdomen CT findings.

Based on the clinical stage and Breslow thickness, a sentinel node biopsy and a wide-margin excision were carried out. On lymphoscintigraphic examination, a major uptake of radionuclide was localized in the popliteal and groin lymph nodes, which were excised and submitted to histopathological examination. These lymph nodes were deemed positive for metastatic melanoma and, consequently, a lymphadenectomy of the deep and superficial groin as well as popliteal regions was indicated. After this procedure, histopathological examination disclosed seven popliteal lymph nodes, all being disease-free.

### Surgical anatomy and the operative technique

Normally, there are 2 to 9 lymph nodes localized within the fat tissue surrounding the nerves and major vessels of the popliteal fossa. Popliteal lymph nodes are deep structures encountered under the investing fascia of the leg, and they receive the lymphatic drainage of the deep structures of the distal leg and foot. Although infrequent, the lymphatic drainage from the skin is direct to these lymph nodes in some individuals. The surgical procedure of popliteal lymph node dissection involves *en bloc* removal of all fibrous, fatty, and lymphatic tissue in the popliteal fossa, with careful preservation of the nerves and vessels.

**Figure 1:**
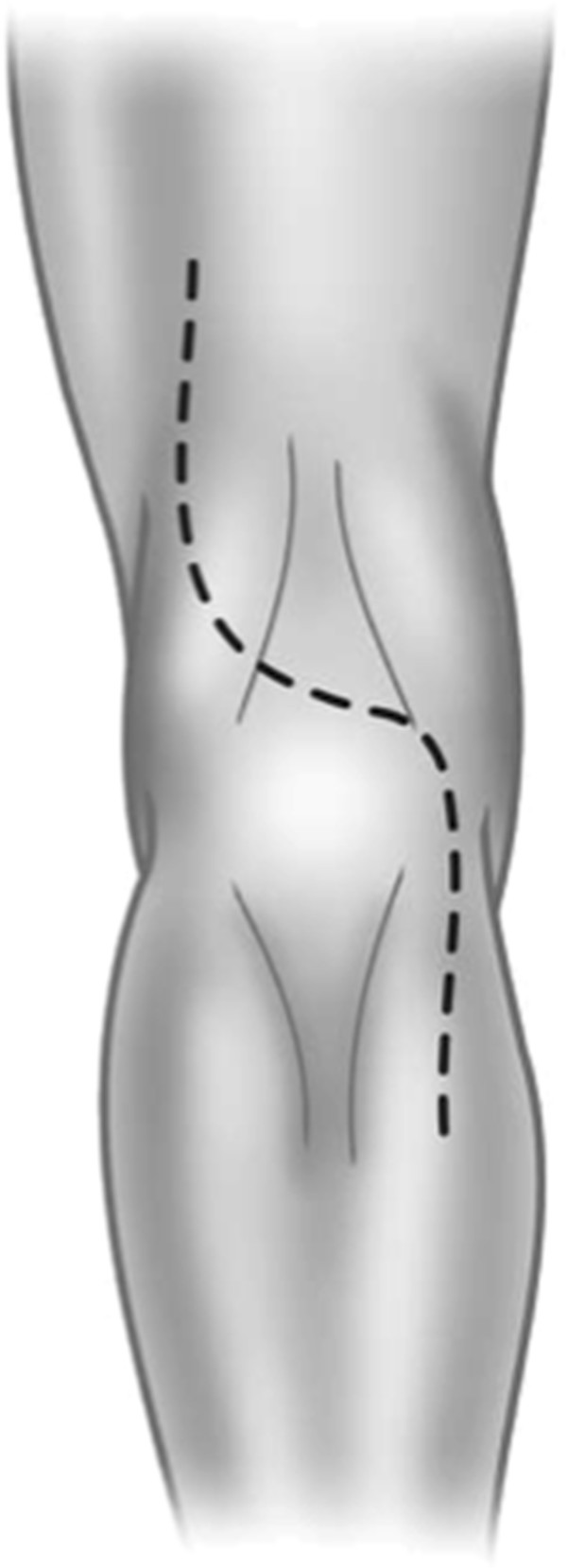
‘S’ shape incision for lymphadenectomy of the popliteal fossa.

The patient is placed in the prone position with the knee slightly flexed. To gain access to the popliteal fossa, an S-shaped incision is performed (Figure 1). The lateral flap is raised first to the surface of the biceps femoris muscle proximally, and the lateral head of the gastrocnemius muscle distally and superficial to the level of Scarpa’s fascia. The medial flap is raised until the semitendinosus and semimembranosus muscle proximally and the medial head of the gastrocnemius muscle distally. Skin hooks or autostatic retractors are used to maintain raised flap traction.

**Figure 2:**
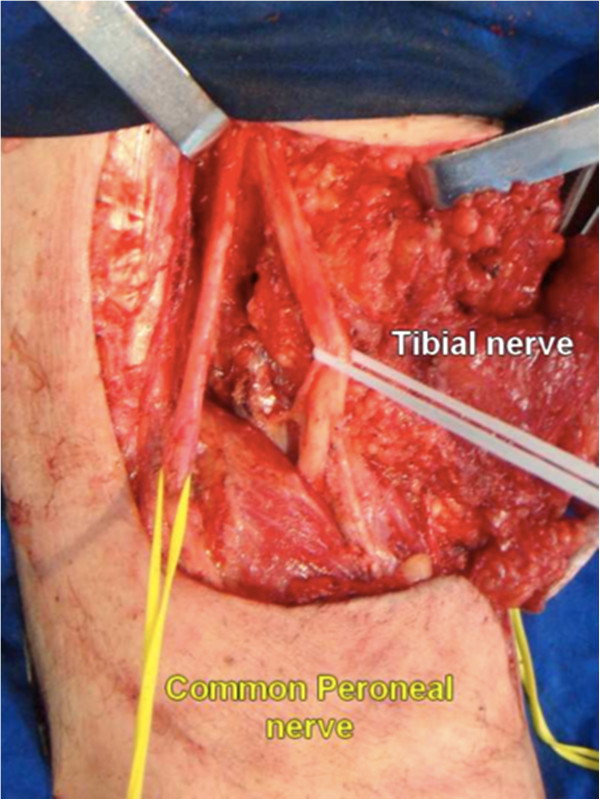
Bifurcation of sciatic nerve into common peroneal nerve and tibial nerve.

The upper angle of the popliteal fossa is formed by the biceps and semimembranosus muscle. Medially to the edge of the biceps femoris muscle, the common peroneal nerve is identified through a layer of fat tissue. An ascending sharp dissection is made superficially to the nerve until the level of its origin from the sciatic nerve. This careful dissection continues distally until the surface of the peroneus longus muscle. At this point, the tibial nerve is also dissected in the popliteal space superficially to the popliteal vessels and both nerves are hooked with vessel loops to allow gentile lateral traction (Figure 2).

**Figure 3:**
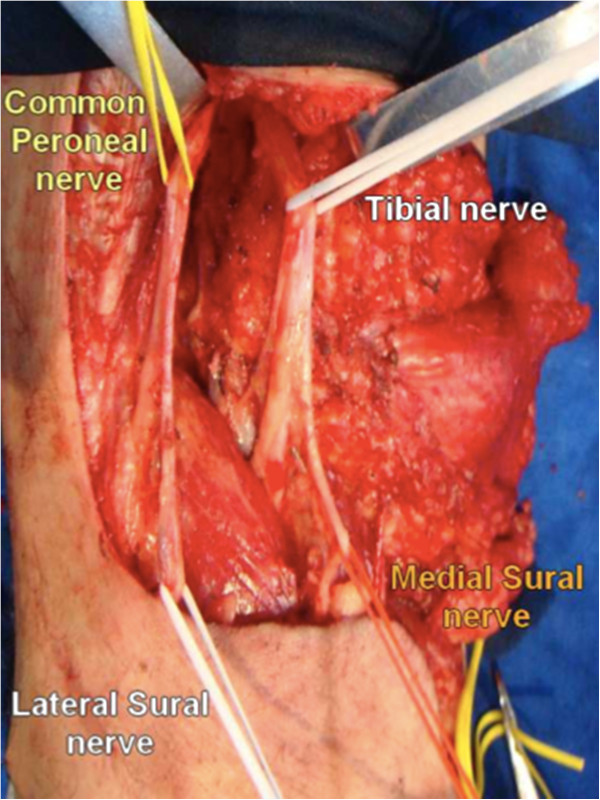
Descending dissection of the major nervous trunks and proper identification of cutaneous sural nerve rami from their origins.

Emerging from the lateral surface of the peroneal nerve, we can identify the lateral sural cutaneous nerve (Figure 3). This nerve is responsible for the sensory innervation to the posterolateral surface of the leg. The sural nerve is formed from the fusion between a communicating ramus of the lateral sural cutaneous nerve and the medial sural cutaneous nerve in the middle of the leg. If possible, this superficial communicating ramus should be preserved during the dissection over the lateral head of the gastrocnemius. In case of tumor involvement of the lateral cutaneous sural nerve or its communicating ramus, their sacrifice does not cause significant sensory deficit, if the medial cutaneous sural nerve is preserved. The medial cutaneous sural nerve has its origin from the tibial nerve and emerges from the popliteal space to run superficially in the groove between the two heads of the gastrocnemius muscle near to the small saphena vein in the middle of the back of the leg. Division of the medial cutaneous sural nerve results in cutaneous anesthesia.

**Figure 4:**
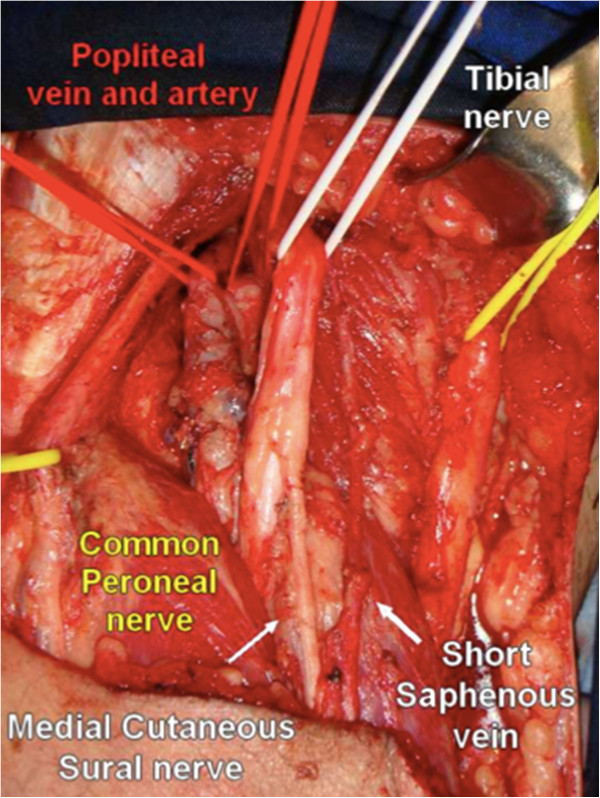
Panoramic view of the popliteal fossa and identification of the major anatomic structures after removal of the surgical specimen.

The deep fascia is stripped off the surface of the gastrocnemius muscle, and the medial cutaneous sural nerve is hooked with vessel loops, like the lateral sural cutaneous nerve, both being held laterally. The popliteal vessels are now approached. The popliteal artery is medial to the popliteal vein and lies deeper. Both are located between the heads of the gastrocnemius muscle (Figure 4).

**Figure 5:**
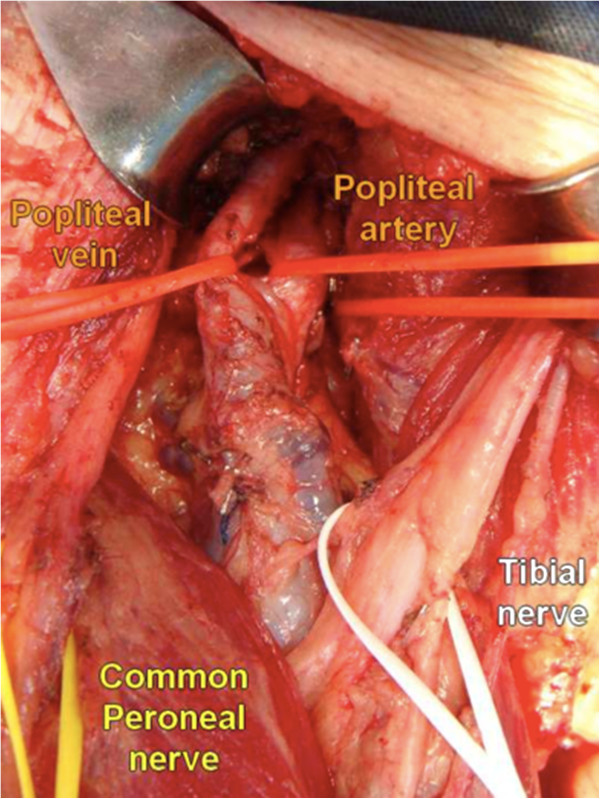
**Close view of the popliteal vessels *in situ*.** Note the popliteal artery lying deeper than the vein in the upper bound of the popliteal fossa.

The descendent dissection continues and the small saphenous vein is ligated close to its insertion in the popliteal vein (Figure 5). All fibrous fatty tissue around the popliteal vessels is removed and some lymph nodes can be identified at this stage. A single lymph node can also be found between the back of the knee and the popliteal artery. Occasionally, there are two popliteal veins and the artery lies between them. The popliteal artery is divided into two major branches: the anterior and posterior tibial artery and a variable number of genicular branches. The surgical specimen is removed in continuity, with a proper identification of the nerves and vessels. As the final step, a closed suction drain is placed in the popliteal fossa.

The superficial lymphatics localized on the calf may accompany the short saphenous vein towards the popliteal fossa draining to popliteal lymph nodes. In a series from the Sidney Cancer Database [3], there were 6.9% direct drainages to one or more popliteal lymph nodes among 236 lymphoscintigrams performed in patients with primary MM at or distal to the knee. Data from these lymphoscintigraphy studies revealed that lymphatic drainage to popliteal lymph nodes can take place anywhere on the leg and foot. However, metastatic disease in popliteal lymph nodes was clinically detectable in 0.31% of the cases only [3]. In a series of Menes et al., popliteal lymph nodes were identified by lymphoscintigraphy in 9% of the 106 patients with MM below the knee or at more distal sites, and metastatic lymph nodes in the popliteal fossa were found in 2.8% of patients [4].

There are two basic indications to perform popliteal lymph node dissection: i) grossly metastatic disease detected on clinical examination or ii) microscopic disease identified in sentinel node lymphadenectomy specimens [5]. Popliteal node dissection is an uncommon procedure but adequately indicated according to data available in current literature [6]. A thorough familiarity of the anatomical landmarks and technical details of the dissection procedure of the popliteal fossa is mandatory.

In 1980, Karakousis published an elegant description of this infrequent operative procedure with detailed information about the surgical anatomy and the technique [2]. Since then, however, there is a paucity of published data on the technical aspects of popliteal lymphadenectomy. To choose the kind of incision, one must consider the optimal exposure necessary to reduce the risk of developing contracture scars. The most frequently used approach of the popliteal fossa by a medial route (described in vascular surgery textbooks to correct popliteal aneurysms or perform femoro-popliteal bypass), does not allow a proper exposure of the structures to accomplish radical lymphadenectomy [7]. The long S-shaped incision used to access the popliteal fossa is designed to offer generous exposure and avoid deforming joint contracture. Other authors prefer a Z-plasty incision for the same purposes [8,9].

To guarantee sufficient exposure, a progressive dissection, initiated by the confection of the lateral flap, was continued throughout removal of all soft tissue. The subsequent surgical steps are followed according to the same principle of removing all fibrous and fatty tissue around, beneath, and alongside the popliteal vessels and nerves. After retraction of the flaps, an easy identification of the peroneal nerve and its ascending dissection until its origin in the sciatic nerve offers prompt identification of the tibial nerve. Subsequent recognition of the sural cutaneous nerves and their branches makes the dissection secure. The next crucial step involves the resection of all fat pad tissue close to the popliteal vessels. Meticulous inspection and palpation of the popliteal fossa must be performed not to leave any lymph node undetected.

## Conclusions

When a surgeon faces a rare surgical problem of regional nodal disease outside the common nodal stations, such as popliteal or epitrochlear chains, the knowledge of anatomy and surgical technique is crucial to guarantee preservation of the key functional structures to avoid permanent disability. Inappropriate dissection can also result in a suboptimal radical lymphadenectomy. The technique of popliteal lymph node dissection described here was successfully performed in a patient with metastatic acral MM, without any post-operative complications or morbidity.

## Consent

Written informed consent was obtained from the patient for publication of this case report and any accompanying images. A copy of the written consent is available for review by the Editor-in-Chief of this journal.

## Competing interests

The authors declare that they have no competing interests.

## Authors’ contributions

FT and EA designed and operated on the case reported. VM and SC made substantial contributions to conception, design, and acquisition of data. ML, TL, GM, and AP wrote the manuscript and edited photographs. SR and EU finally reviewed and actively edited the manuscript. All authors read and approved the final manuscript.
